# Mitochondrial morphology and mtDNA content in fibroblasts from patients with different types of mucopolysaccharidosis

**DOI:** 10.3389/abp.2026.16345

**Published:** 2026-06-22

**Authors:** Klaudia Walczak, Lidia Gaffke, Karolina Pierzynowska, Natalia Sowa-Rogozińska, Ewa Piotrowska

**Affiliations:** 1 Department of Molecular Biology, University of Gdańsk, Gdańsk, Poland; 2 Department of Physiology, Medical University of Gdańsk, Gdańsk, Poland

**Keywords:** fluorescence microscopy, mitochondria, mitochondrial DNA, MPS, mucopolysaccharidosis

## Abstract

Mucopolysaccharidosis (MPS) is a group of inherited metabolic diseases, characterized by defects in the degradation of glycosaminoglycans and their accumulation in lysosomes. However, various secondary cellular changes also contribute to the pathomechanism of MPS. Previous studies have reached contradictory conclusions about the changes in mitochondria in MPS, from increased numbers of mitochondria to impaired activities of some mitochondrial respiratory chain enzymes to no changes in mitochondrial respiration. In this preliminary, hypothesis-generating study, mitochondrial network morphology and mitochondrial DNA (mtDNA) abundance were investigated in fibroblasts derived from patients suffering from diverse MPS types. Fluorescence microscopy and real-time PCR were used to estimate these parameters, respectively. No significant changes in the mitochondrial network morphology were detected in MPS fibroblasts relative to control cells. Decreased levels of mtDNA relative to nuclear DNA levels were evident in some (I, II, IIIA, IIID, and VI) but not all MPS types compared to control fibroblasts. The results of this study suggest that there are some, although perhaps not dramatic, impairments of mitochondrial functions in some MPS types; however, they do not provide direct evidence of mitochondrial dysfunction. Therefore, these findings should be interpreted as descriptive and exploratory, highlighting the need for further functional and mechanistic studies.

## Introduction

Mucopolysaccharidoses (MPSs) are a group of lysosomal storage disorders (LSDs) caused by mutations affecting genes that encode enzymes involved in the catabolism of glycosaminoglycans (GAGs). The intralysosomal accumulation of undegraded GAGs, including heparan sulfate, dermatan sulfate, chondroitin sulfate, and keratan sulfate, which leads to the enlargement of these organelles, has historically been considered the main, if not the only, explanation for MPS pathophysiology. However, recent advancements in our understanding of cellular processes and pathways have revealed a growing body of evidence linking lysosomal swelling and dysfunction to the impairment of other cellular systems, including mitochondrial dysregulation (Plotegher and Duchen, 2017; Fecarotta et al., 2020; Leal et al., 2022).

Mitochondria, being essential for energy production and cellular metabolism, are increasingly recognized for their role in the pathophysiology of LSDs (de la Mata et al., 2016; Plotegher and Duchen, 2017; Stepien et al., 2020; Stepien et al., 2022). A link between lysosomal dysfunction and impaired mitochondrial dynamics and activity has also been suggested in MPSs. Electron microscopy analysis of neurons in MPS IIIA and MPS IIIC murine models has revealed a relatively increased number of mitochondria (Settembre et al., 2008; Martins et al., 2015). Additionally, mitochondria in MPS IIIC neurons have been found to exhibit varied shapes and structural abnormalities, such as swelling and disorganization of the inner membrane, in addition to significantly lower activities of certain mitochondrial respiratory chain enzymes (Martins et al., 2015). However, studies on an MPS II cellular model (Jacques et al., 2023) and liver cells from a mouse model (Pinheiro et al., 2024) did not provide evidence of changes in mitochondrial respiration; instead, they suggested alterations in redox homeostasis and oxidative DNA damage. The detailed characterization of mitochondrial network morphology and mitochondrial DNA (mtDNA) content in patient-derived cells remains underexplored. Therefore, this study aimed to address this gap by quantifying mitochondrial particles, assessing mitochondrial network area per cell, and determining mtDNA content in fibroblasts derived from patients with different MPS types.

## Materials and methods

### Cell lines and culturing conditions

Fibroblasts from MPS patients were purchased from the Coriell Institute, while control human dermal fibroblasts, adult (HDFa), were purchased from Sigma-Aldrich. All cell lines are characterized in Table 1. The cells were cultured in DMEM (Gibco) supplemented with 10% FBS (Gibco) and 1% Antibiotic-Antimycotic (Gibco), under standard conditions, as previously described (Gaffke et al., 2020; Pierzynowska et al., 2020).

**TABLE 1:** Characteristics of the fibroblast cell cultures used in this study.

Provider/Catalogue ID	Description	Mutated gene, chromosomal location	Mutation*	Sex*	Age at sampling (years)*	Race/ethnicity*	Passages used in this study
CI/GM00798	MPS I	*IDUA*, 4p16.3	Homozygote p.Trp402Ter/p.Trp402Ter	Female	1	White	3–10
CI/GM13203	MPS II	*IDS*, Xp28	Hemizygote p.His70ProfsTer29	Male	3	White/Haitian	11–18
CI/GM00879	MPS IIIA	*SGSH*, 17q25.3	Complex heterozygote p.Glu447Lys/p.Arg245His	Female	3	White	9–16
CI/GM00156	MPS IIIB	*NAGLU*, 17q21	Homozygote p.Arg626Ter/p.Arg626Ter	Male	7	White	9–15
CI/GM05157	MPS IIIC	*HGSNAT*, 8p11.1	N/D	Male	8	N/D	7–14
CI/GM05093	MPS IIID	*GNS*, 12q14	Homozygote p.Arg355Ter/p.Arg355Ter	Male	7	Asiatic Indian	6–12
CI/GM00593	MPS IVA	*GALNS*, 16q24.3	N/D	Female	7	White/Mexican	7–14
CI/GM03251	MPS IVB	*GLB1*, 3p22.3	Complex heterozygote p.Trp273Leu/p.Trp509Cys	Female	4	White	8–13
CI/GM03722	MPS VI	*ARSB*, 4q14.1	N/D	Female	3	Black/African American	5–8
CI/GM00121	MPS VII	*GUSB*, 7q21.11	Complex heterozygote p.Trp627Cys/p.Arg356Ter	Male	3	Black/African American	11–17
CI/GM17494	MPS IX	*HYAL1*, 3p21.3	N/D	Female	14	N/D	4–10
SA/106-05A	HDFa - control	N/A	N/A	N/D	N/D	N/D	8–14

* According to the provider’s description; CI- coriell institute; SA- Sigma-Aldrich; N/D-no data; N/A-not applicable.

### Fluorescence microscopy

Fibroblasts (5 × 10^4^ cells) were seeded onto uncoated glass coverslips (20 mm diameter) in 12-well plates and allowed to attach overnight. The next day, the medium was removed, and mitochondria were stained with MitoTracker Green FM (Thermo Fisher Scientific) at 300 nM for 45 min at 37 °C. Subsequently, the cells underwent three washes with prewarmed PBS and were fixed with 2% paraformaldehyde. The fixed cells were then incubated in PBS containing 0.2% Triton X-100 for 15 min, followed by five washes with PBS. Coverslips were mounted on glass slides with a mounting medium.

The slides were imaged using a fluorescence microscope (Leica DMI4000B) with a ×100 objective and identical acquisition settings for all samples. Image analysis was performed using ImageJ software. Identical thresholding parameters (default 2D threshold settings) were applied to all the images, and the same analysis pipeline was used for all samples. Image analysis was performed in a blinded manner.

The total mitochondrial area and the number of mitochondrial network particles per cell were determined by manually outlining the positively labeled structures. The number of mitochondrial network particles was defined as the number of discrete fluorescent objects identified after thresholding MitoTracker-positive structures in ImageJ. These objects represent fragments of the mitochondrial network rather than individual mitochondria.

This analysis was performed on 15 cells per line, derived from two technical replicates across three different passages. Individual cells were treated as observational units in the analysis.

### DNA extraction and measurement of mtDNA content

Each cell line was cultured in triplicate for DNA isolation. A QIAamp DNA Mini Kit (Qiagen) was used to extract total cellular DNA, according to the manufacturer’s instructions. DNA concentration and purity were measured by NanoDrop 2000. Relative mtDNA copy number was determined by real-time quantitative PCR using LightCycler 480 SYBR Green I Master (Roche). The following primers were used for amplification: mitochondrial 16S rDNA (forward: 5′-CGA​AAG​GAC​AAG​AGA​AAT​AAG​G-3′; reverse: 5′-CTG​TAA​AGT​TTT​AAG​TTT​TAT​GCG-3′; amplicon size: 152 bp; annealing temperature: 53 °C) and nuclear β-globin (forward: 5′-CAA​CTT​CAT​CCA​CGT​TCA​CC-3′; reverse: 5′-GAA​GAG​CCA​AGG​ACA​GGT​AC-3′; amplicon size: 268 bp; annealing temperature: 60 °C). The primer sequences were selected based on previously published protocols (Jędrak et al., 2017). Relative mtDNA was calculated using the ΔCt method mtDNA vs. nuclear DNA), and the results are presented as the mtDNA/nDNA ratio.

### Statistical analysis

Statistical analyses were conducted using GraphPad Prism 10.1.2. The tests utilized are specified in the figure legends.

Microscopy-based measurements were conducted on individual cells; therefore, the results should be interpreted as descriptive, as individual cells do not fully represent independent biological replicates. No outlier exclusion was performed; all analyzed cells were included in the statistical analysis.

## Results

### Mitochondrial network characteristics in MPS fibroblasts do not differ significantly from those of the control

**FIGURE 1:**
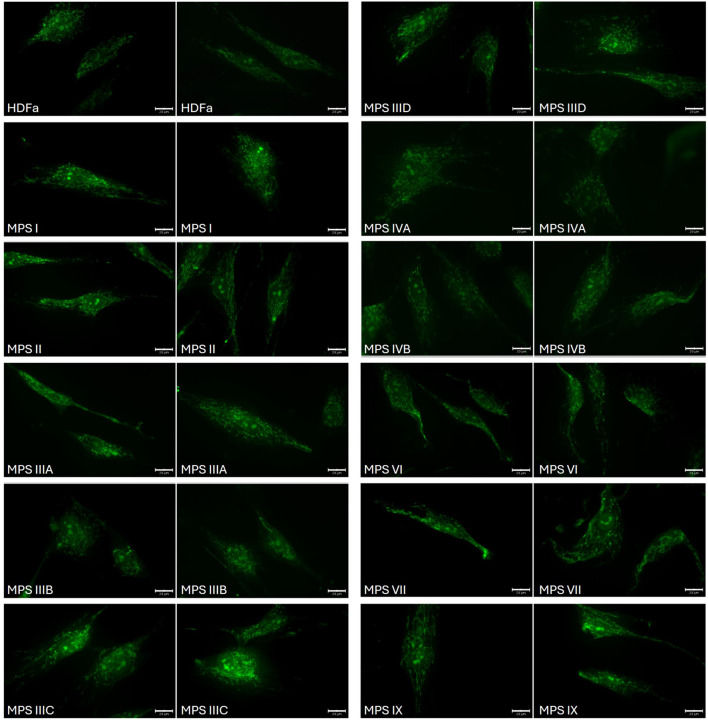
Representative images of MitoTracker Green-labeled mitochondria in analyzed fibroblasts. The scale bars correspond to 20 µm. HDFa indicates the control cell line, and the other symbols depict different types of MPS.

For each of the 11 MPS cell lines and the control HDFa line, counts of mitochondrial network particles and measurements of mitochondrial area were conducted in 15 cells, totaling 180 cells analyzed (example fluorescent images of the fibroblasts used in this analysis are shown in Figure 1).

**FIGURE 2:**
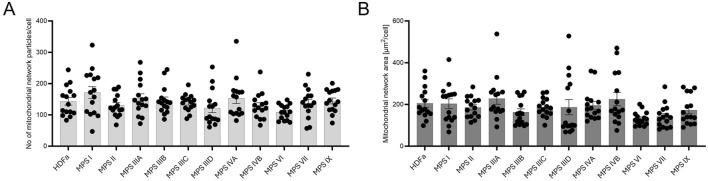
MPS fibroblast mitochondrial network characteristics. **(A)** Number of mitochondrial network particles per cell. **(B)** Area of mitochondrial network [µm^2^/cell]. For each cell line, MitoTracker-labeled structures were analyzed in n = 15 cells (two to three cells from two technical replicates from three different passages). HDFa indicates the control cell line, and the other symbols depict different types of MPS. The bars and whiskers represent the means and standard errors of the mean, respectively. No statistical significance (p > 0.05) was found between cell lines (one-way ANOVA).

The number of mitochondrial network particles in the analyzed cells ranged from 47 to 335, with the lowest average number (109) observed in MPS VI and the highest (171) in MPS I (Figure 2A). Fibroblasts from patients with MPS II, IIIC, IIID, IVB, VI, and VII presented lower average numbers of mitochondrial network particles compared to the control HDFa, while MPS I, IIIA, IIIB, IVA, and IX had higher averages. However, none of these differences were statistically significant; thus, we conclude that there are no considerable differences in this parameter between MPS and control fibroblasts.

The mitochondrial network area per cell ranged from 68.10 to 537.73 μm^2^, with the lowest average area (132.25 μm^2^) observed in MPS VI fibroblasts and the highest (228.05 μm^2^) observed in MPS IIIA (Figure 2B). The average area of the mitochondrial network was larger than that of control healthy cells only in MPS IIIA and IVB fibroblasts; in the other MPS types, it was smaller. Again, however, no statistical significance was found between cell lines, indicating a lack of considerable differences in the mitochondrial network area between MPS fibroblasts and control cells derived from healthy individuals.

### Certain types of MPS fibroblasts have a decreased mtDNA/nDNA relative copy number

**FIGURE 3:**
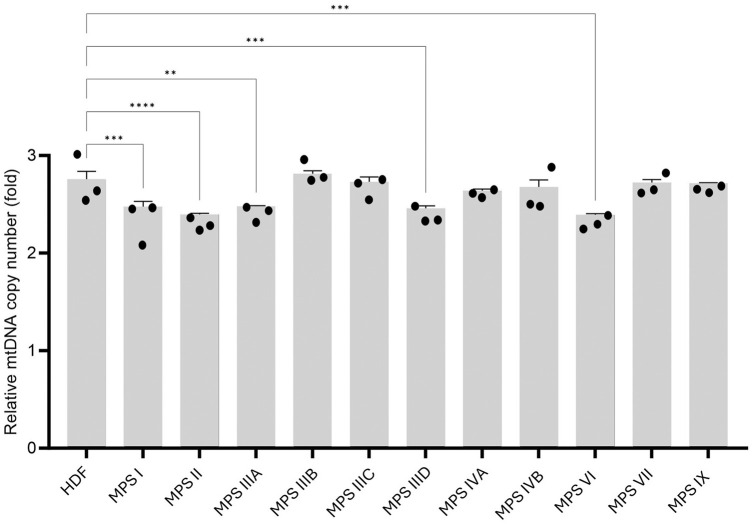
Relative mtDNA copy number in MPS fibroblasts. The bars and whiskers represent the mean and standard error of the mean from n = 3 independent experiments, respectively. HDFa indicates the control cell line, and the other symbols depict different types of MPS. The asterisks indicate values that are significantly different from the controls (HDFa cells): **p < 0.01, ***p < 0.001, and ****p < 0.0001. A one-way ANOVA Dunnett test was used.

Fibroblast mtDNA levels were evaluated using real-time quantitative PCR to assess the ratio of mtDNA to nuclear DNA (nDNA), with the 16S rDNA gene as the target mitochondrial gene and the beta-globin-encoding gene as the reference nuclear gene. The relative number of mtDNA copies in MPS cells was comparable to the control (in MPS IIIB, IIIC, IVA, IVB, VII, and IX), but decreased in MPS I, II, IIIA, IIID, and VI, as shown in Figure 3.

## Discussion

As demonstrated in various LSDs, lysosomal dysfunction is linked to impaired autophagy, leading to compromised mitophagy (Plotegher and Duchen, 2017). Consequently, dysfunctional mitochondria can accumulate. Since morphological changes are relatively easily identifiable through fluorescence imaging microscopy, this technique was utilized in our study to quantify mitochondrial particles (structures that respond with a positive MitoTracker Green signal) and to assess the mitochondrial network area in fibroblasts from patients with different types of MPS.

Although mitochondrial morphology is highly diverse and dynamic, changing across different cell types, within individual cells, and in response to various cellular conditions (Chang and Reynolds, 2006), no significant differences were observed between MPS and control fibroblasts in the analyzed parameters. This is in contrast to previous electron microscopy analyses of individual mitochondria in the same cell lines, which revealed not only an increased number but also a narrowed and elongated shape of these organelles in MPS IIID, IVA, IVB, VII, and IX fibroblasts (Gaffke et al., 2021). One possible explanation for this inconsistency is that the parameter “number of mitochondrial network particles” reflects the number of fluorescent objects identified after image threshold-based segmentation, and thus does not necessarily indicate isolated singular mitochondria, but rather elements of the mitochondrial network. Since mitochondria form a highly interconnected and dynamic network, a single “particle” may represent a fragment of this network rather than a single organelle.

Additionally, MitoTracker localizes to mitochondria regardless of mitochondrial membrane potential and therefore does not distinguish between functionally active and impaired organelles. This represents an important limitation of the present study, as mitochondrial bioenergetic dysfunction, which is in many instances related to mitochondrial fragmentation or elongation, is often associated with changes in membrane potential. Future studies should therefore incorporate membrane potential-sensitive probes (e.g., TMRE or TMRM) and direct functional assays, including measurements of respiration, ATP production, reactive oxygen species (ROS), and mitophagy.

Mitochondria produce energy through oxidative phosphorylation, a process that generates reactive oxygen species (ROS) as a byproduct. Consequently, mitochondrial dysfunction can lead to imbalanced ROS levels, which can damage biomolecules, including DNA. Recognizing that disturbed mtDNA content in cells can indicate mitochondrial dysfunction, we evaluated mtDNA levels in MPS fibroblasts. The most pronounced decrease in mtDNA copy number compared to the healthy controls was observed in MPS II fibroblasts, followed by a slight decrease in MPS I, IIIA, IIID, and VI fibroblasts. In the other examined MPS cell lines, mtDNA copy number did not differ from the control.

Importantly, the observed decrease in mtDNA copy number without corresponding changes in morphology-based parameters may reflect alterations in mitochondrial biogenesis or mtDNA maintenance rather than changes in mitochondrial mass or network organization. Indeed, mtDNA content can vary independently of mitochondrial morphology and is known to be influenced by factors such as oxidative stress, replication efficiency, and cellular metabolic state (Guantes et al., 2016; Malik and Czajka, 2013).

When analyzing the results of this study, and to assess some contradictions in previously reported results (mentioned above), one should consider that mtDNA copy number varies across different tissues. However, the mechanisms that regulate tissue-specific mtDNA copy numbers are not fully understood. Additionally, the mtDNA copy number is not fixed and can vary significantly, with population studies showing a two-to tenfold variation in a particular tissue among individuals, which is even mirrored in the clinical range for “normal” mtDNA content, defined as 40%–150% of the average for a given population (Kozhukhar et al., 2021). Therefore, the differences observed in this study should be interpreted with caution, particularly given the use of a single donor-derived fibroblast line per MPS subtype, which does not allow for the separation of disease-specific effects from donor-specific variability.

A further limitation of our study is the use of skin fibroblasts as a model system. Although skin changes are recognized symptoms of MPS, and fibroblasts are widely used due to their accessibility and sustainability in culture (Daré et al., 2026; Olesen et al., 2022; Suarez et al., 2026), skin fibroblasts may not fully recapitulate tissue -specific aspects of MPS pathophysiology, particularly in the most affected organs, such as the central nervous system.

In addition, the microscopy-based analysis relied on individual cells and observational units, which may introduce a risk of pseudoreplication. Therefore, the results should be interpreted as descriptive rather than reflecting fully independent biological replication.

Taken together, our findings should be interpreted as hypothesis-generating, highlighting potential mitochondrial alterations that require confirmation in more comprehensive functional studies. This study should therefore be considered a preliminary, signal-generating investigation aimed at identifying potential trends across multiple MPS subtypes rather than providing a comprehensible mechanistic characterization.

## Conclusions

Mitochondrial network morphology, as assessed by fluorescence microscopy-based parameters, does not differ significantly between fibroblasts derived from patients with various types of mucopolysaccharidosis and control cells. In contrast, mtDNA levels are decreased in certain types of MPS (I, II, IIIA, IIID, and VI).

These findings suggest potential alterations in mitochondrial homeostasis in certain MPS subtypes; however, they do not provide direct evidence of mitochondrial dysfunction. Therefore, the results should be viewed as preliminary and interpreted with caution and hypothesis-generating, highlighting the need for further functional studies to clarify the role of mitochondria in MPS pathophysiology.

## Data Availability

The raw data supporting the conclusions of this article will be made available by the authors, without undue reservation.
